# Close the High Seas to Fishing?

**DOI:** 10.1371/journal.pbio.1001826

**Published:** 2014-03-25

**Authors:** Crow White, Christopher Costello

**Affiliations:** 1 Department of Biological Sciences, California Polytechnic State University, San Luis Obispo, California, United States of America; 2 Bren School of Environmental Science and Management, University of California, Santa Barbara, Santa Barbara, California, United States of America

## Abstract

The world's oceans are governed as a system of over 150 sovereign exclusive economic zones (EEZs, ∼42% of the ocean) and one large high seas (HS) commons (∼58% of ocean) with essentially open access. Many high-valued fish species such as tuna, billfish, and shark migrate around these large oceanic regions, which as a consequence of competition across EEZs and a global race-to-fish on the HS, have been over-exploited and now return far less than their economic potential. We address this global challenge by analyzing with a spatial bioeconomic model the effects of completely closing the HS to fishing. This policy both induces cooperation among countries in the exploitation of migratory stocks and provides a refuge sufficiently large to recover and maintain these stocks at levels close to those that would maximize fisheries returns. We find that completely closing the HS to fishing would simultaneously give rise to large gains in fisheries profit (>100%), fisheries yields (>30%), and fish stock conservation (>150%). We also find that changing EEZ size may benefit some fisheries; nonetheless, a complete closure of the HS still returns larger fishery and conservation outcomes than does a HS open to fishing.

## Abstract 2

Closing the high seas to fishing could induce cooperation among countries and lead to global increases in fish stocks, harvest, and profit.

## Introduction

**Figure 1:**
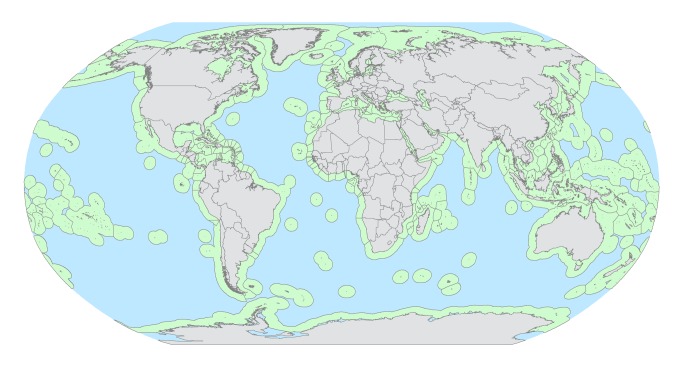
Global map of exclusive economic zones (green) and high seas (blue) oceanic areas.

The past 60 years have been a tumultuous period for the world's marine fisheries. In the early 1950s few stocks had been exploited heavily; but without explicit governance, large industrial fisheries took hold and systematically overexploited many stocks [1],[2]. In 1994 the United Nations Convention on the Law of the Sea (UNCLOS) implemented Exclusive Economic Zones (EEZs) adjacent to all coastal nations (Figure 1). These property rights extend 200 nm (∼42% of the ocean) and allow countries to exclude foreign fleets and exclusively manage fisheries within their jurisdictions [3],[4]. Indeed, for countries with science-based fisheries management policies, many local stocks and fisheries contained in their EEZs are rebuilding [5]–[7]. But for many pelagic, migratory stocks such as tuna, billfish, and shark, the size of the EEZs has been insufficient to incentivize sustainable fishing behavior [8]–[10]. Fish that traverse multiple EEZs and the high seas ([HS], ∼58% of ocean) are overexploited relative to those contained in a single EEZ [11],[12].

This observation accords with two long-standing theoretical predictions: First, that open access tends to produce a “tragedy of the commons” (an unregulated state of affairs in which individuals inefficiently compete for a shared, limited resource, resulting in its eventual ruin [13]), where fishermen will race to fish, drive stocks down, and compete away economic value [12],[14]. Thus, we may expect HS stocks to be overexploited. Second, that spatial property rights, such as EEZs, will mediate overexploitation, but only to the extent that they enclose the full range of the species [15],[16]. If fish migrate [17],[18] and/or have dispersive larvae [19], the ensuing spatial externality presents a competitive situation in which countries act like players in a non-cooperative game [20]. Thus, we may expect that the more EEZs a stock traverses, the less likely a sustainable outcome. When put together, these theories suggest that migratory species pose perhaps the greatest global challenge to sustainable fisheries management [9].

In an ideal world, all nations would cooperate in the management of transboundary stocks. Indeed for decades hundreds of attempts have been made at multi-lateral agreements primarily through regional fishery management organizations, which aim to coordinate fishing across EEZs and on the HS. While some exceptions exist, these efforts are widely regarded as a failure [12],[16],[21],[22].

## Modeling High Seas Closure

It is within this context that we analyze the alternative of a complete HS closure. While large marine protected areas (MPAs) in the HS are of increasing interest [23]–[25], a complete closure has not been proposed, and what little analysis exists suggests there would be substantial economic losses from such a policy [26]. Smaller MPAs, increasingly common and well-studied in coastal waters [27],[28], are too small to produce significant benefits for most migratory stocks [26]. Also, closing only a portion of the high seas may simply displace fishing effort to other open-access areas [29], thereby leaving the problem unsolved. Instead, a complete closure of the HS may simultaneously achieve three desirable outcomes: (1) It acts as a coordination mechanism across EEZs; (2) it reduces overall exploitation rates; and (3) it protects a sufficient range of the stock to allow rebuilding.

The “risk” of closing the HS is that some species may not range sufficiently far into EEZs, leaving those stocks underexploited. Therefore, we also consider changing the size of the EEZs. A key aspect of our analysis involves modeling the behavioral competition between countries for stocks in EEZs and on the HS. To do so, we adopt a game theoretical perspective (for estimating the strategic decisions among interacting players in a competitive scenario), and use coupled biological-economic models in which stocks traverse multiple EEZs and the HS and the relevant players compete for fisheries profits. Thus, rather than making assumptions about exploitation rates we derive the likely behavioral adjustments under any given policy.

We model a large range of governance and biological scenarios that represent the range of conditions for pelagic, migratory species in the world's oceans. Any given scenario is defined by: (1) the fraction of the fish stock's range (and fishery) in EEZs (the remainder being on the HS); (2) the number of EEZs traversed by the stock; (3) the biological parameters of the stock; and (4) the degree of site fidelity of individual fish. For each scenario we evaluate three states of governance of the HS: open access (“HS open [OA]”), closed to fishing (“HS closed”), or competed for by *N* players (“HS open [*N*]”). We use a widely used cost function throughout. Our baseline model adopts conservative parameter values, stacking the deck against a HS closure. As a benchmark we also model the idealistic case of complete global cooperation across the entire range of the stock. Full methods are given in Text S1.

We examined the effects of a HS closure first with a simple example. Suppose a reasonably fast-growing stock (*r* = 0.2) [30] has high site-fidelity (*S* = 0.75), and is proportionally distributed across the HS (58%) and ten EEZs (42%). Our model predicted that when the HS were open, the ten countries would compete on both the HS and their EEZs, and drive stocks to a third of the economically optimal stock size. When the HS were closed, countries would compete across EEZs, but no fishing would occur on the HS: stock increased everywhere (4-fold on the HS and 30% in EEZs), profit more than doubled, and yield increased by 42% (though profit and yield are still only 68% and 84% of their theoretical values under complete cooperation). The disproportionate increase in profit is due to interacting effects of elimination of the inefficient overexploitation on the HS, enhanced coordination across EEZs incentivized by the spillover and protection of fish from the HS, and reduced fishery cost from harvesting a higher stock density in the EEZs. Collectively, these factors raise profit (and yield) beyond the loss from not fishing on the HS.

**Figure 2:**
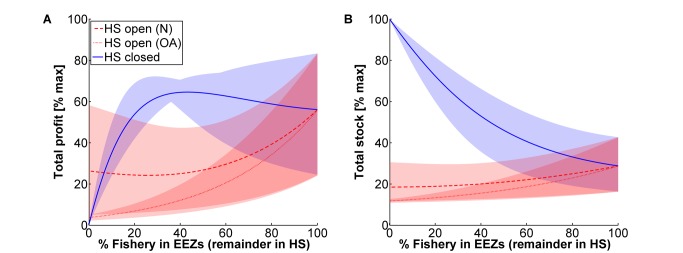
Fishery and conservation value as a function of high seas policy. Total fishery profit (A) and fish stock (B), as a percentage of the maximum possible, in relation to percentage of the fishery's geographic distribution that is within EEZs (i.e., in territorial waters, with the remainder in the HS), under alternative policies for managing the HS (see legend). Lines indicate the baseline scenario (*N* = 10, *r* = 0.2, *S* = 0.75, *C* = 1), and shading the minimum and maximum values across the factorial evaluation of *N* = 5–50 and *r* = 0.1–0.3.

The figures plot various results against the fraction of the fishery contained in EEZs. When a fishery is mostly in EEZs, the problem boiled down to a transboundary one—where an international fish stock was not contained in any one country's jurisdiction. In that case, closing the HS did not, by itself, fix coordination problems across nations (Figures 2A and S2), because escaped stocks still could be harvested by a competing fishery [9]. Instead, if a fishery is primarily on the HS, closing the HS eliminated the fishery, generating a loss. For fisheries targeting pelagic, migratory stocks, typically some but not all of the fishery occurs in EEZs [8],[17],[18],[31],[32]. In those cases, closing the HS nearly always benefited the fishery: with our baseline parameters, if at least 10% of the fishery were contained in EEZs, then closing the HS increased fishery profits (Figures 2A and S2A). Across the full range of parameters, if at least 20% of the fishery were contained in EEZs, then closing the HS increased profit. The explanation is simple: most species harvested on the HS are vulnerable to overexploitation when the HS are fished, but are likely to recover (and benefit sovereign fisheries via spillover) when the HS are closed. As expected, profits and yields from a HS closure were never as large as levels achievable under complete global cooperation of harvest levels across the HS and EEZs (at best, they were on average ∼60% and ∼80% as high, occurring when ∼40% of the fishery is in EEZs; Figures 2A and S1). Regarding conservation, a HS closure always resulted in large increases in fish stocks (possibly by >100%; Figures 2B and S2C), consistent with the literature cataloging the conservation benefits of marine reserves [28] (but see [29],[33] for counter-examples, particularly in relation to cumulative impacts and management challenges in marine ecosystems).

**Figure 3:**
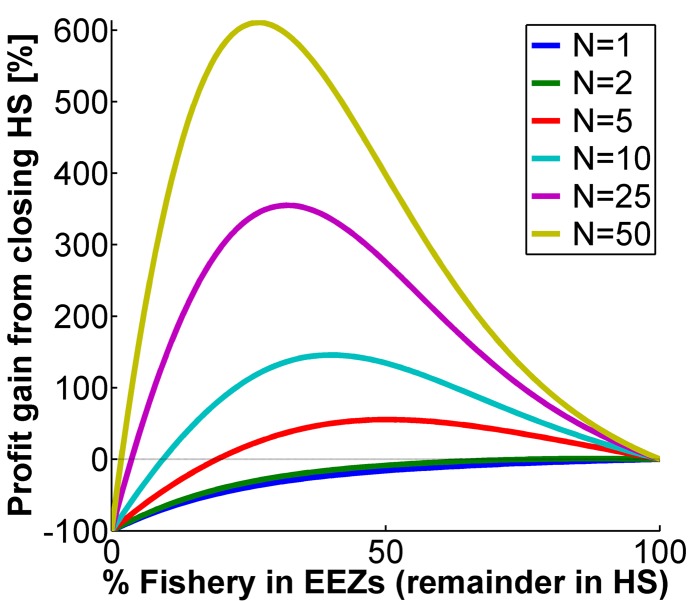
Fishery gain as a function of EEZ number. Percentage gain in total fishery profit from closing the HS in relation to percentage of the fishery's geographic distribution that is within EEZs and number of EEZs that the fishery species' range transverses (*N*; see legend). Gains are with respect to profits under HS open (*N*); for gains with respect to HS open (OA) see Figure S3. Baseline values *r* = 0.2, *S* = 0.75, and *C* = 1. Horizontal dotted line is for reference indicating zero gain.

The more EEZs traversed by the stock (*N*) the worse was the tragedy of the commons, and the greater was the percentage increase from a HS closure (Figures 3 and S3). Under typical values of *N* (say, *N* = 10–20), the gain was considerable. If fish are evenly distributed between HS and EEZs, so 42% are enclosed in EEZs, then any *N*>3 scenario provided benefits, and any *N*>10 more than doubled the value of the fishery. In the extreme, for stocks that traverse 50 or more EEZs, the gains could exceed 500%. If the true *N* is large (say, *N* = 20), but nations cooperate, the effective *N* may be small (say, *N* = 5). Even in that case, a HS closure increased fishery profit. We assumed relatively high site fidelity (*S* = 0.75); results were strengthened under lower site fidelity (Figure S4). All of the above results held over a large range of growth rates, though gains from HS closure were largest for slower-growing species (Figure S5).

Holding a species' range constant, larger EEZs will increase the fraction of the fishery contained in EEZs (rightward shift in Figures 2–3 and S1, S2, S3, S4, S5, S6). Focusing on the blue and red shading in Figure 2, except for narrow EEZs, closing the HS typically generated large gains for both profit and stock (also see Figure S2). Further, when the HS are open, the worst possible EEZ width was around 40%—this width gave rise to the lowest profit and stock of any possible configuration of EEZs (on average, around 25% and 20% of what was possible for each). Thus the status quo (open HS and 42% in EEZs) was nearly the worst case scenario: the HS are heavily overexploited and countries' EEZs are too small to protect stocks from non-cooperative harvest. Why not simply extend the EEZs [16]? Doing so entailed a benefit, but as the EEZs enlarge, the source of overharvest changed from being primarily a HS problem to being primarily a transboundary problem. Rather, we have shown that completely closing the HS to fishing provided ample protection to the migratory stocks from transboundary overharvest, and without changing EEZ size still allowed each country sufficient space to harvest profitably in their EEZ.

## From Modeling to Implementation

While our main finding is likely to hold across many, if not all, ocean basins, there inevitably will be distributional impacts. For example, the handful of countries whose current fishing fleets specialize in fishing the HS (e.g., Japan, China, and Spain [31]) may be harmed by the closure. On the other hand, these countries' HS losses may be offset by enhanced fishing opportunities in their EEZs as stocks rebuild. Developing countries whose stocks are depleted by HS over-exploitation but who have not invested in HS fleets may benefit most from a HS closure. Thus, for a HS closure to be considered in practice, it will be important for future work to explore empirically the fishery and country-level distributional impacts of this proposal.

While a complete policy analysis is beyond our scope, a few comments are worth noting. Closing the HS to fishing may seem politically unviable, partly because UNCLOS recognizes the freedom to fish there by all nations [3]. However, UNCLOS also requires ecosystem protection and equitable and efficient utilization of the ocean's resources. Thus, there is demand for a new legal instrument for HS governance [34],[35]; it could support a HS closure to meet the UNCLOS equity, economic, and conservation objectives. First, gains from a HS closure are attributable to fish spillover into EEZs, thus although not fishing in the HS, the freedom to fish resources from the HS is maintained. Second, the closure may only apply to mobile fishery species (and perhaps over-exploited by-catch species), and not sessile species (*S* = 1) where fishery value would be reduced (Figure S4). Third, a portion of the gains from closing the HS could be distributed among land-locked nations in a fashion similar to existing transfers for transboundary fisheries [36]. Finally, although perfect compliance with a HS closure may not be necessary for gains to emerge (Figure S6), enforcement is a concern [8],[25]. Yet major advances in fishery surveillance technology [23], recent increases in the scope and use of agreements on the HS (including with MPAs) [8],[23],[25],[37],[38], and perhaps part of the fishery gains due to the HS closure, could be used to support its enforcement. Research on the viability of these options would contribute substantially to our understanding of the political and economic feasibility of closing the high seas.

## Supporting Information

Figure S1 **Fishery value as a function of high seas policy.** Total fishery yield, as a percentage of the maximum possible, in relation to percentage of the fishery's geographic distribution that is within EEZs (remainder in the high seas), under alternative policies for the high seas (see legend). Lines indicate the baseline scenario (*N* = 10, *r* = 0.2, *S* = 0.75, *C* = 1), and shading the minimum and maximum values across the factorial evaluation of *N* = 5–50 and *r* = 0.1–0.3.
(PDF)

Figure S2 **Gain from closing the high seas.** Percentage gain in total fishery profit (A), yield (B), and stock (C) from closing the high seas in relation to percentage of the fishery's geographic distribution that is within EEZs (remainder in the high seas). Gains are calculated with respect to outcomes under alternative policies for the high seas open (see legend). Lines indicate the baseline scenario (*N* = 10, *r* = 0.2, *S* = 0.75, *C* = 1), and shading the minimum and maximum values across the factorial evaluation of *N* = 5–50 and *r* = 0.1–0.3. Horizontal dotted lines are for reference indicating zero gain.
(PDF)

Figure S3 **Gain as a function of EEZ number.** Percentage gain in total fishery profit (A, B), yield (C, D), and stock (E, F) from closing the high seas in relation to percentage of the fishery's geographic distribution that is within EEZs (remainder in the high seas), and the number of EEZs that the fishery transverses (*N*; see legend). Gains are calculated with respect to outcomes under HS open (*N*) (left panels) and HS open (OA) (right panels) policies, using baseline values *r* = 0.2, *S* = 0.75, and *C* = 1. Horizontal dotted lines are for reference indicating zero gain.
(PDF)

Figure S4 **Gain as a function of local site fidelity.** Percentage gain in total fishery profit (A, B), yield (C, D), and stock (E, F) from closing the high seas in relation to percentage of the fishery's geographic distribution that is within EEZs (remainder in the high seas), and the level of enhanced local site-fidelity (*S*; see legend). *S* = 0 indicates “common pool” redistribution of fish in relation to relative patch area. *S* = 1 indicates no movement of fish among patches. Gains are calculated with respect to outcomes under HS open (*N*) (left panels) and HS open (OA) (right panels) policies, using baseline values *N* = 10, *r* = 0.2, and *C* = 1. Horizontal dotted lines are for reference indicating zero gain.
(PDF)

Figure S5 **Gain as a function of intrinsic growth rate.** Percentage gain in total fishery profit (A, B), yield (C, D), and stock (E, F) from closing the high seas in relation to percentage of the fishery's geographic distribution that is within EEZs (remainder in the high seas), and the intrinsic growth rate of the fishery species (*r*; see legend). Gains are calculated with respect to outcomes under HS open (*N*) (left panels) and HS open (OA) (right panels) policies, using baseline values *N* = 10, *S* = 0.75, and *C* = 1. Horizontal dotted lines are for reference indicating zero gain.
(PDF)

Figure S6 **Gain as a function of compliance.** Percentage gain in total fishery profit (A, B), yield (C, D), and stock (E, F) from closing the high seas in relation to percentage of the fishery's geographic distribution that is within EEZs (remainder in the high seas), and the level of compliance with the high seas closure (*C*; see legend). *C* = 0 indicates fishing effort in the high seas is equivalent to the level under HS open (*N*). *C* = 1 indicates no fishing in the high seas. Gains are calculated with respect to outcomes under HS open (*N*) (left panels) and HS open (OA) (right panels) policies, using baseline values *N* = 10, *r* = 0.2, and *S* = 0.75. Horizontal dotted lines are for reference indicating zero gain.
(PDF)

Text S1 **Methods.**
(PDF)
