# Supplementary figures and images for: Close the High Seas to Fishing?
Source: PLoS Biol. 2014 Mar 25;12(3):e1001826. doi: 10.1371/journal.pbio.1001826 (PMC3965379; doi:10.1371/journal.pbio.1001826)

# Close the High Seas to Fishing?

Crow White and Christopher Costello

Figure S1

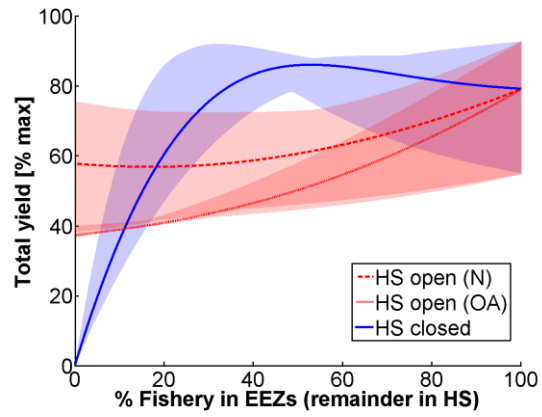

Supplement: Figure S1 — Fishery value as a function of high seas policy. Total fishery yield, as a percentage of the maximum possible, in relation to percentage of the fishery's geographic distribution that is within EEZs (remainder in the high seas), under alternative policies for the high seas (see legend). Lines indicate the baseline scenario (N = 10, r = 0.2, S = 0.75, C = 1), and shading the minimum and maximum values across the factorial evaluation of N = 5–50 and r = 0.1–0.3. (PDF) [file pbio.1001826.s001.pdf]

# Close the High Seas to Fishing?

Crow White and Christopher Costello

Figure S2

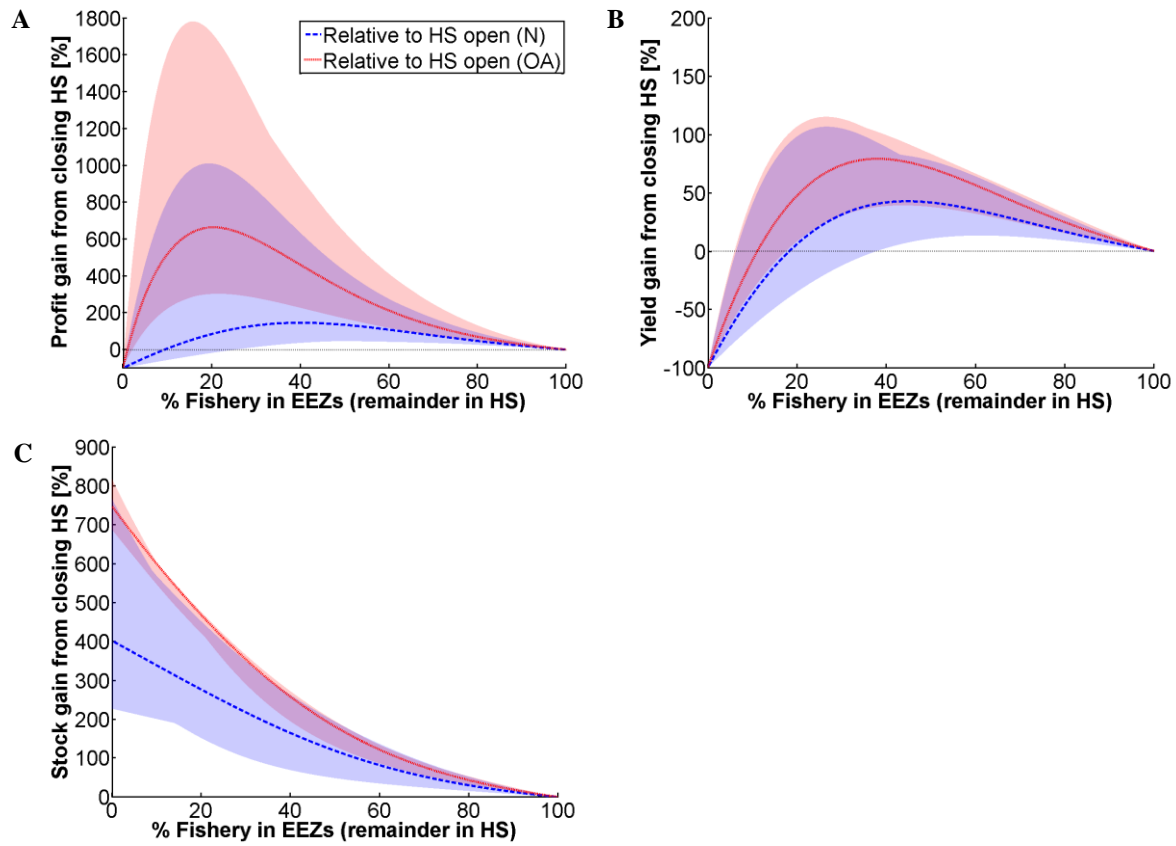

Supplement: Figure S2 — Gain from closing the high seas. Percentage gain in total fishery profit (A), yield (B), and stock (C) from closing the high seas in relation to percentage of the fishery's geographic distribution that is within EEZs (remainder in the high seas). Gains are calculated with respect to outcomes under alternative policies for the high seas open (see legend). Lines indicate the baseline scenario (N = 10, r = 0.2, S = 0.75, C = 1), and shading the minimum and maximum values across the factorial evaluation of N = 5–50 and r = 0.1–0.3. Horizontal dotted lines are for reference indicating zero gain. (PDF) [file pbio.1001826.s002.pdf]

# **Close the High Seas to Fishing?** Crow White and Christopher Costello

**Figure S3**

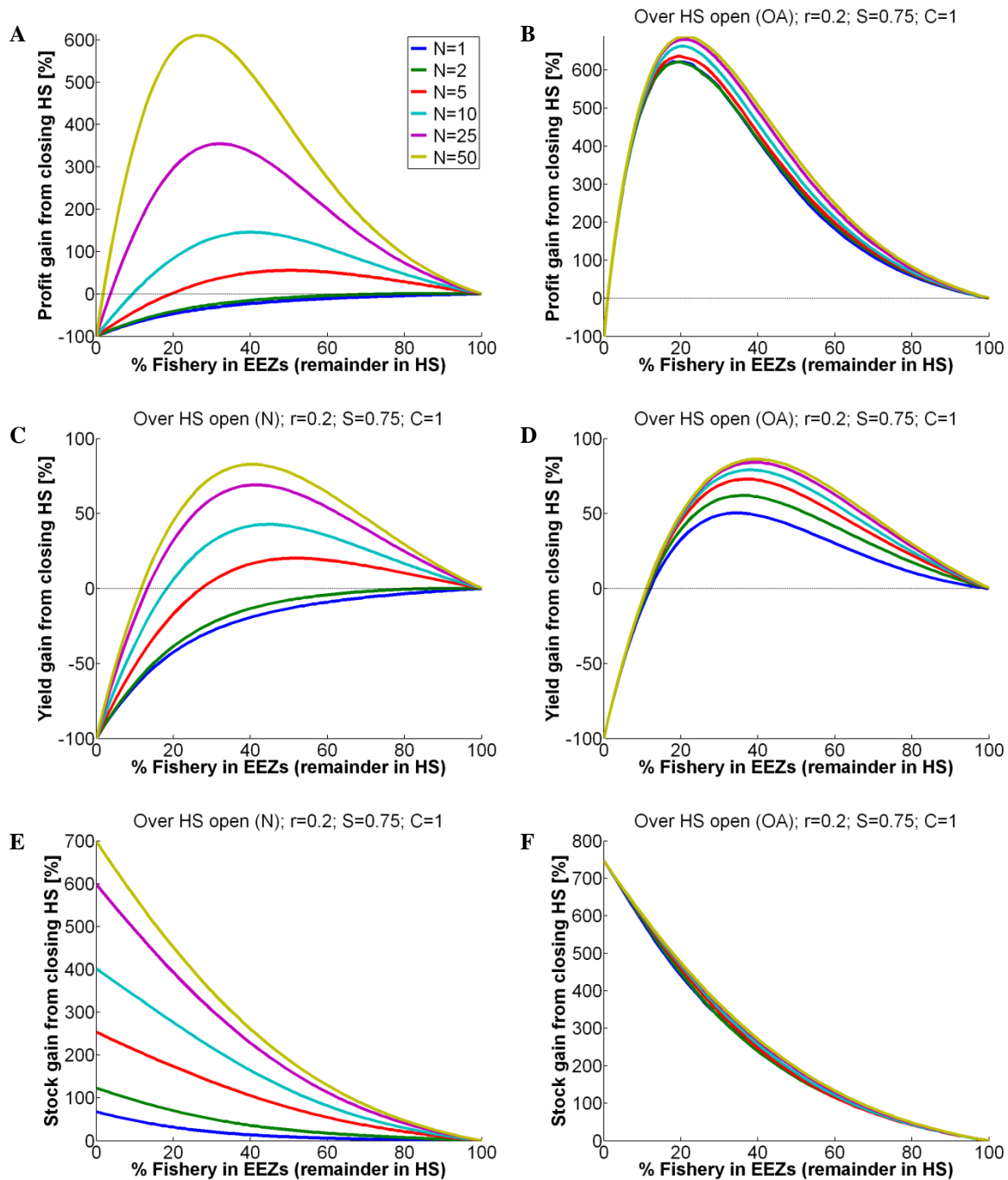

Supplement: Figure S3 — Gain as a function of EEZ number. Percentage gain in total fishery profit (A, B), yield (C, D), and stock (E, F) from closing the high seas in relation to percentage of the fishery's geographic distribution that is within EEZs (remainder in the high seas), and the number of EEZs that the fishery transverses (N; see legend). Gains are calculated with respect to outcomes under HS open (N) (left panels) and HS open (OA) (right panels) policies, using baseline values r = 0.2, S = 0.75, and C = 1. Horizontal dotted lines are for reference indicating zero gain. (PDF) [file pbio.1001826.s003.pdf]

# **Close the High Seas to Fishing?** Crow White and Christopher Costello

**Figure S4**

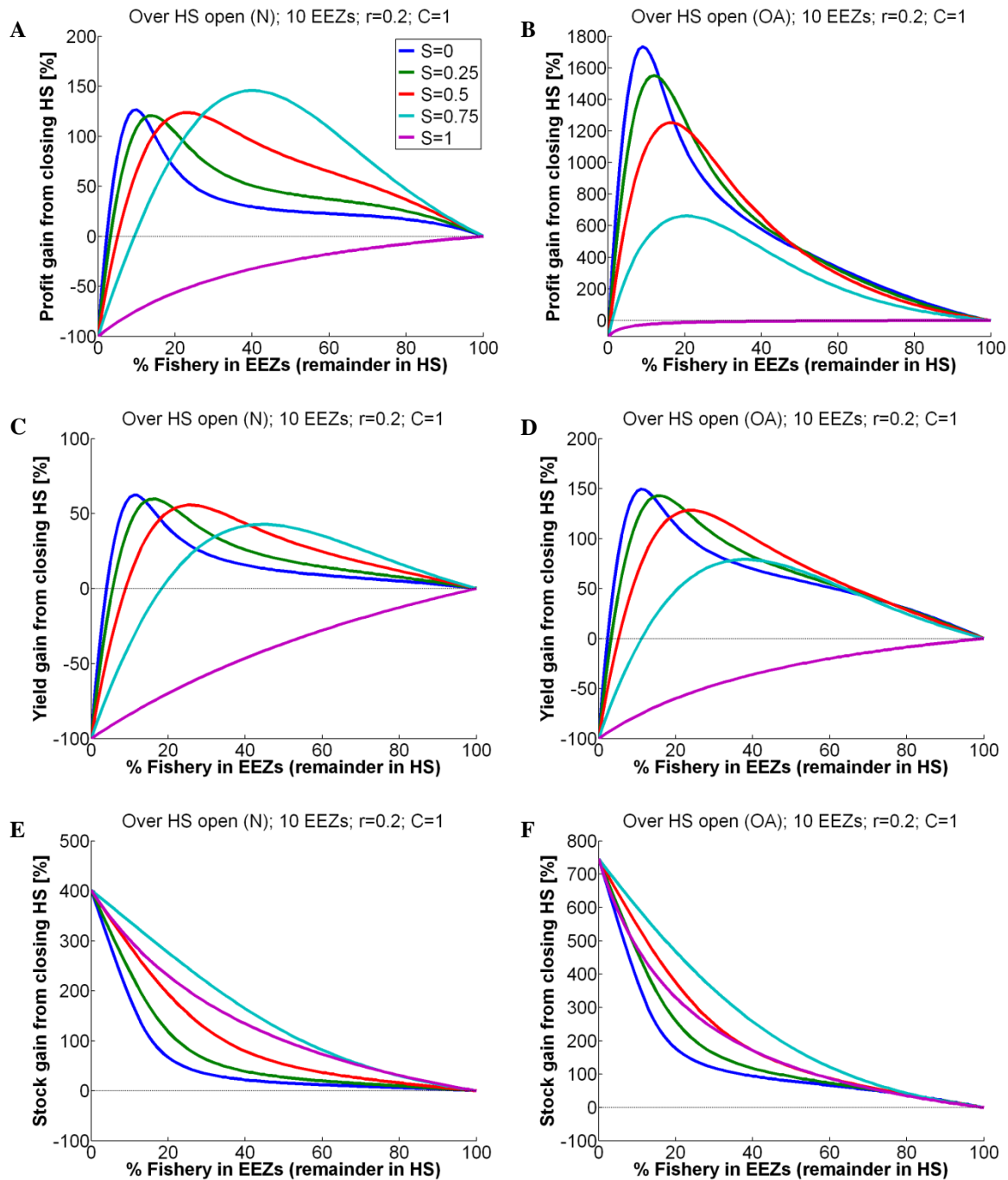

Supplement: Figure S4 — Gain as a function of local site fidelity. Percentage gain in total fishery profit (A, B), yield (C, D), and stock (E, F) from closing the high seas in relation to percentage of the fishery's geographic distribution that is within EEZs (remainder in the high seas), and the level of enhanced local site-fidelity (S; see legend). S = 0 indicates “common pool” redistribution of fish in relation to relative patch area. S = 1 indicates no movement of fish among patches. Gains are calculated with respect to outcomes under HS open (N) (left panels) and HS open (OA) (right panels) policies, using baseline values N = 10, r = 0.2, and C = 1. Horizontal dotted lines are for reference indicating zero gain. (PDF) [file pbio.1001826.s004.pdf]

# **Close the High Seas to Fishing?** Crow White and Christopher Costello

**Figure S5**

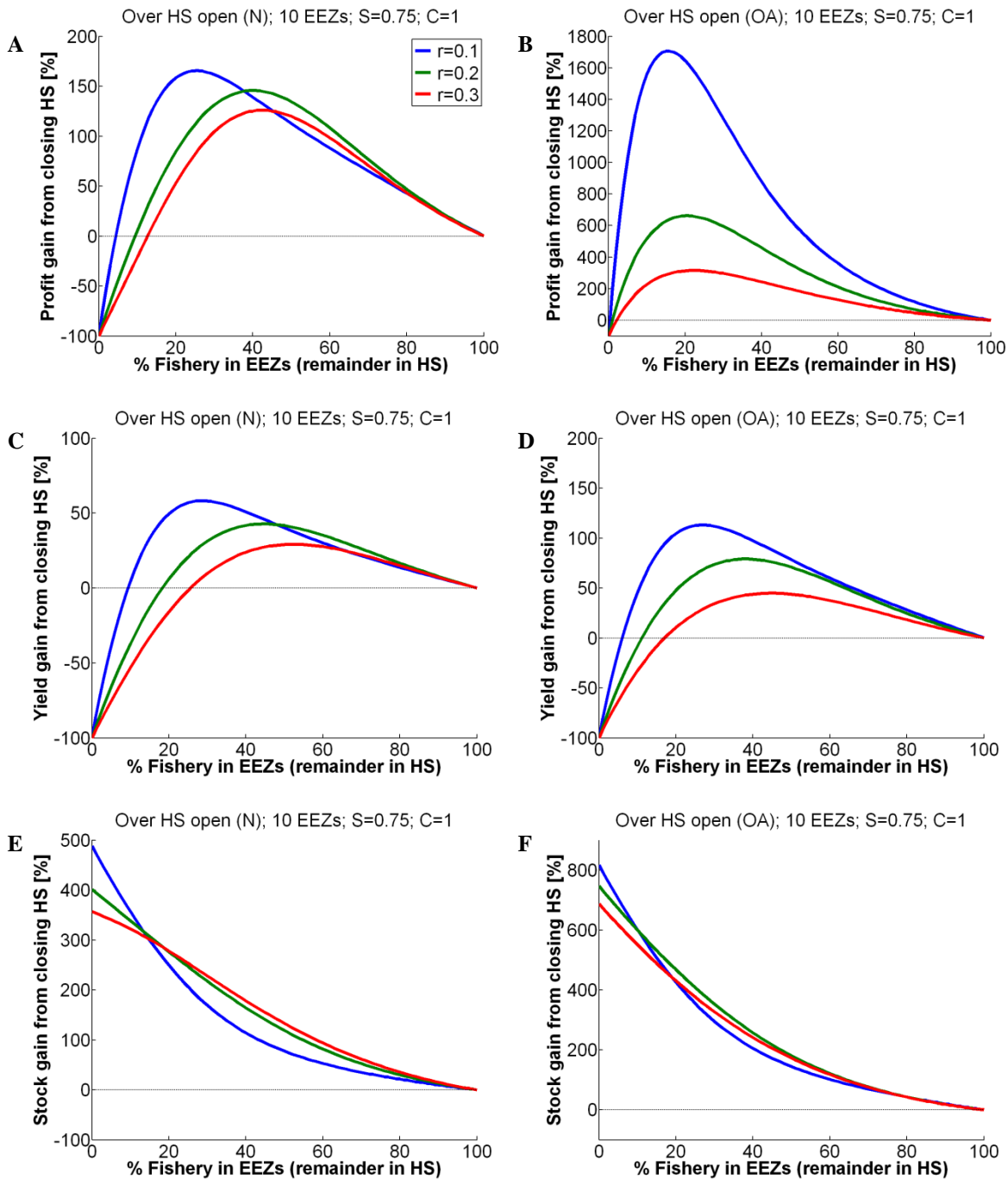

Supplement: Figure S5 — Gain as a function of intrinsic growth rate. Percentage gain in total fishery profit (A, B), yield (C, D), and stock (E, F) from closing the high seas in relation to percentage of the fishery's geographic distribution that is within EEZs (remainder in the high seas), and the intrinsic growth rate of the fishery species (r; see legend). Gains are calculated with respect to outcomes under HS open (N) (left panels) and HS open (OA) (right panels) policies, using baseline values N = 10, S = 0.75, and C = 1. Horizontal dotted lines are for reference indicating zero gain. (PDF) [file pbio.1001826.s005.pdf]

# **Close the High Seas to Fishing?** Crow White and Christopher Costello

**Figure S6**

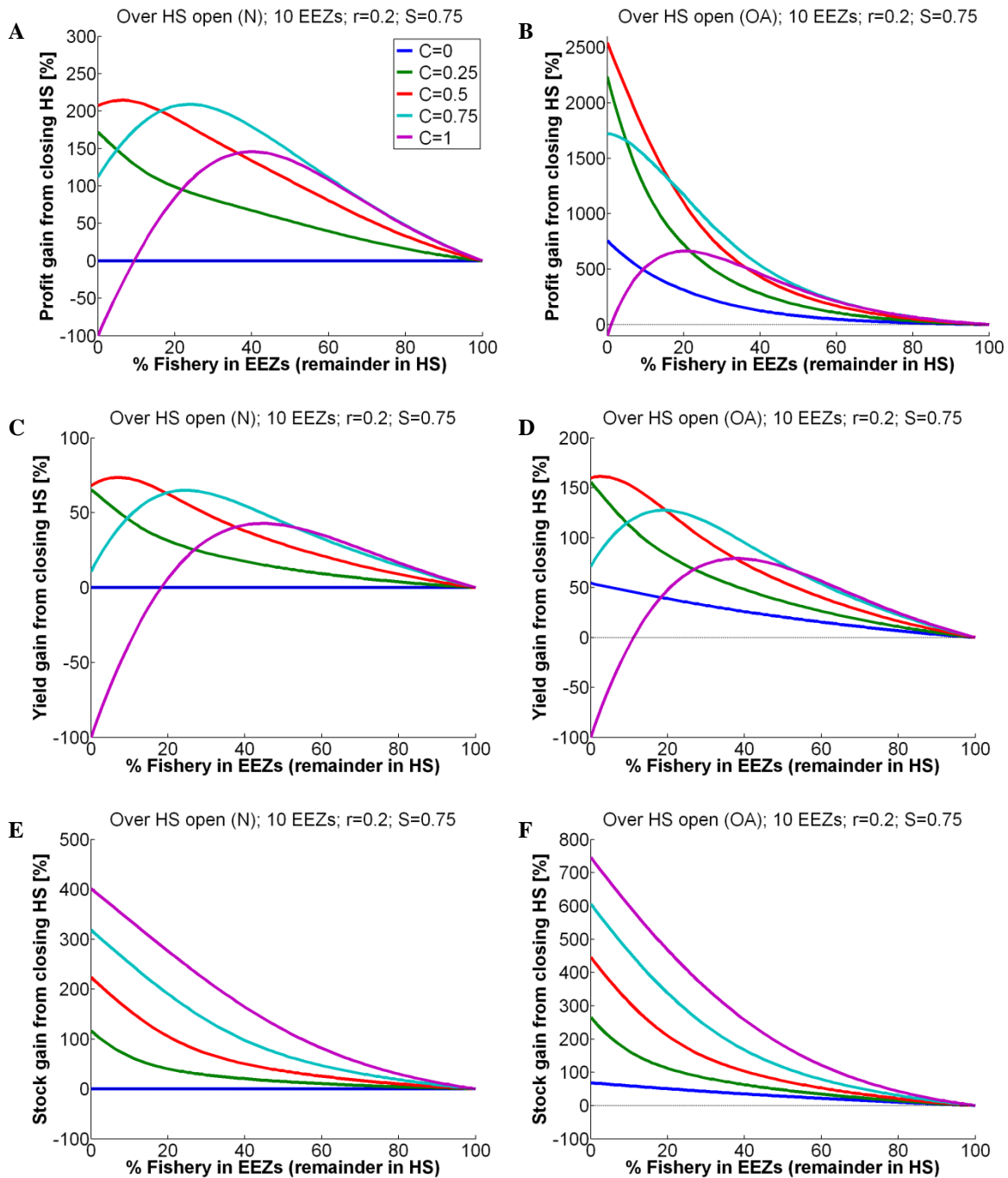

Supplement: Figure S6 — Gain as a function of compliance. Percentage gain in total fishery profit (A, B), yield (C, D), and stock (E, F) from closing the high seas in relation to percentage of the fishery's geographic distribution that is within EEZs (remainder in the high seas), and the level of compliance with the high seas closure (C; see legend). C = 0 indicates fishing effort in the high seas is equivalent to the level under HS open (N). C = 1 indicates no fishing in the high seas. Gains are calculated with respect to outcomes under HS open (N) (left panels) and HS open (OA) (right panels) policies, using baseline values N = 10, r = 0.2, and S = 0.75. Horizontal dotted lines are for reference indicating zero gain. (PDF) [file pbio.1001826.s006.pdf]
