# Supplementary material for: Close the High Seas to Fishing?
Source: PLoS Biol. 2014 Mar 25;12(3):e1001826. doi: 10.1371/journal.pbio.1001826 (PMC3965379; doi:10.1371/journal.pbio.1001826)
Supplement: Text S1 — Methods. (PDF) [file pbio.1001826.s007.pdf]

Close the High Seas to Fishing?

Crow White and Christopher Costello

SUPPLEMENTARY INFORMATION – METHODS S1

Methods Summary

We simulated alternative policies using a spatial logistic fish population model coupled with a game theoretic fishery economic model. Fish stock is observed and then harvested in *N* EEZ and one HS patches, generating a residual stock that grows and disperses among the patches. Different combinations of *N* and proportion of the species' range in EEZs versus HS represent alternative geopolitical and biogeographic scenarios. Fish movement among patches is represented by a dispersal kernel with enhanced local site fidelity. Fishery profit in a patch is a function of revenue from harvest (yield) and market price and cost of fishing in relation to local fishing effort and stock density. Solutions were obtained by solving for the game theoretic Nash equilibrium among *N* players (EEZ nations traversed), where each player simultaneously chooses the (set of) harvest effort level(s) that maximizes profit, taking as given the effort levels by the other players. Two HS open scenarios were considered: one that limits harvest in the HS to *N* players ("HS open (*N*)"), and one that allows open access HS ("HS open (OA)"). In the latter, the HS represent a true commons and the stock is harvested down to a density where marginal profit is zero. Taking this as given, each of the *N* players chooses a harvest effort level for its EEZ that maximizes the player's profit, given the other players' decisions. In HS open (*N*), each player has two choice variables, harvest effort in its EEZ and in the HS, that are determined simultaneously among all players, and HS profit is distributed proportionally to the players' HS effort levels. Under the HS closed scenario players are limited to choosing effort levels in the EEZs, except when non-compliance was considered. In that case, the EEZ players exhibit positive efforts in the HS, up to a maximum level equal to what they exhibited in HS open (*N*). Taking as given the level of HS closure (non-)compliance, the players then choose harvest effort levels in their EEZs to maximize profit. Full Methods details are given below.

Methods Details

There are N+1 patches: N EEZs and 1 high seas (HS). By convention, we assumed that patch $N+1$ is the high seas. The equation of motion in patch i is:

$$x_{i,t+1} = \frac{\sum_{j=1}^{N+1} D_{ji} A_j g\left(e_{j,t}\right)}{A_i} \,. \qquad (1)$$

The timing is thus: the present period stock density in each patch $\left(x_{j,t}\right)$ is observed and then harvested $\left(h_{j,t}\right)$ giving residual stock density $\left(e_{j,t}\right)$, which then grows $\left(g\left(e_{j,t}\right)\right)$, and, following conversion to stock abundance (via multiplication by patch area, A_j), disperses across the system $\left(D_{ji}\right)$. The resulting stock abundance is divided by patch area, A_i, to indicate stock density at the

beginning of the subsequent period $(x_{i,t+1})$. The system is divided into fraction Z in the high seas and fraction (1-Z) that is divided equally among the N EEZs. Thus each EEZ represents fraction $Q=(1-Z)/N$ of the system. The entire system is set to area of 1, thus A_i=Z for the high seas patch and A_i=Q for each EEZ patch.

Harvest (i.e., yield) is measured in units of biomass abundance and is calculated in relation to stock density, habitat area and harvest effort, E_i, in each patch:

$$h_{i,t}=x_{i,t}A_i f(E_i), \tag{2}$$

where $f(E_i)$ is the fraction of stock harvested and is an increasing function of harvest effort. Residual stock density, or density of escapement from harvest, is thus:

$$e_{i,t}=x_{i,t}(1-f(E_i)). \tag{3}$$

Patch-i harvesters earn price p_i per unit harvest, and marginal harvest cost is a decreasing function of resource stock density in patch i. Given $h_{i,t}$ harvest in patch i, profit in the patch is:

$$\Pi_{i,t}=p_i h_{i,t}-A_i\int_{e_{i,t}}^{x_{i,t}}c_i(s)ds, \tag{4}$$

where $c'(s)<0$ (i.e., higher resource stock density reduces per-unit harvest cost).

Parameters

a) Harvest

We used the exponential survival function for calculating harvest and escapement:

$$f(E_i)=1-e^{-E_i q}, \tag{5}$$

where q is a catchability coefficient modulating effort's effect on harvest and, without loss of generality, is set to 1.

b) Growth

We represented growth of the stock using the discrete-time logistic function [Schaefer logistic model; 1]:

$$g(e_i)=e_i+re_i(1-e_i/K), \tag{6}$$

which is the same across all patches. We set stock carrying capacity to K=1 unit biomass density. For representing tuna and other large-bodied marine fish typically the focus of pelagic high seas fisheries we used intrinsic population growth rates r = 0.1-0.3 [2].

c) Dispersal

Dispersal was based a "common pool" model that we modified to consider enhanced site fidelity (i.e., self-recruitment). The fishery species traverses N EEZs and the high seas. With no enhanced site fidelity, dispersal depends only on the proportional areas in the system; in that case the dispersal matrix is:

$$\mathbf{D} = \begin{bmatrix} Q & \dots & Q & Z \\ Q & \dots & Q & Z \\ \dots & \dots & \dots & \dots \\ Q & \dots & Q & Z \end{bmatrix}, \qquad (7)$$

where a row-column cell value represents the fraction of dispersal from a source to destination patch (see Equation 1); each row sums to 1 thus the system is closed. Thus, for example, given 42% of a species' range in N=10 EEZs and the remainder in the high seas (i.e., Z=0.58), fish self-recruitment rates are $D_{EEZ,EEZ}$ = 4.2% in each EEZ and $D_{HS,HS}$ = 58% in the high seas. Fishery species exhibit varying degrees of site fidelity [3-5]. With enhanced site fidelity, the fractions of self-recruitment (i.e., diagonal cells in **D**) are increased from the Q (EEZ) and Z (high seas) values in equation (7), up to a maximum of 1 (i.e., 100% self-recruitment). Concurrently, the off-diagonal cells in each row (i.e., fractions of cross-recruitment) counterbalance the increase in self-recruitment with a commensurate decrease that is distributed among the destination patches (columns) in proportion to their area. Thus enhanced self-recruitment is balanced by reduced cross-recruitment and the rows in **D** still sum to 1. Enhancement of self-recruitment is governed by the parameter S, where $0 \le S \le 1$. This gives rise to the following dispersal matrix:

$$\mathbf{D} = \begin{bmatrix} Q+(1-Q)S & Q-(1-Q)S(Q/(1-Q)) & \dots & Z-(1-Q)S(Z/(1-Q)) \\ Q-(1-Q)S(Q/(1-Q)) & Q+(1-Q)S & \dots & Z-(1-Q)S(Z/(1-Q)) \\ \dots & \dots & \dots & \dots \\ Q-(1-Z)S(Q/(1-Z)) & Q-(1-Z)S(Q/(1-Z)) & \dots & Z+(1-Z)S \end{bmatrix}. \qquad (8)$$

If $S=0$, enhanced self-recruitment is removed and we revert to the common pool dispersal kernel (i.e., equation (7)). If $S=1$, we have 100% site fidelity; no dispersal occurs out of any patch. For all of the figures in the main text and SI (except Fig. S5, where we show effects of $0 \le S \le 1$) we set S=0.75, representing conservatively high self-recruitment rates within each patch. Thus, for example, the baseline scenario N=10 EEZs, Z=0.58 and S=0.75 indicates self-recruitment rates of $D_{EEZ,EEZ}$ = 76% in each EEZ and $D_{HS,HS}$ = 90% in the high seas.

d) Economics

Price per unit harvest was set to p = 1. Marginal cost of harvest follows the "stock effect" function, $c_i(s) = \theta/s$ [6,7], where the scaling parameter was set to 10% of the carrying capacity density of the stock, θ = 0.1K. Below this stock density marginal cost exceeds price and it is unprofitable to harvest. All else equal, this cost function suggests that a completely open access global fishery would harvest the stock down to 10% of its virgin biomass level. Although they

exist in practice, we do not consider economic subsidies to fisheries (e.g., by their home country). Subsidies are expected to generate an increase in fishery harvest rate because they contribute to fishery revenue and thus enable them to profitably drive stocks to lower densities [8].

e) Number of players

Each EEZ represents a single player in the decision-theoretic game. Each player chooses a harvest effort level in their corresponding EEZ, and, depending on the management scenario, possibly in the high seas. Profit in an EEZ goes exclusively to that EEZ player, while profits from harvesting the high seas are distributed among the players in proportion to their high seas harvest effort levels. We considered a range of players, N=1-50, to reflect the fact that some marine fishery species have wide ranging distributions and other do not, as well as the fact that some coastal areas contain many small EEZs while other areas contain fewer larger EEZs.

Solution approach

We focused on steady-state economic payoffs. Solutions were obtained by solving for the Nash equilibrium of the game among the N players, where each player simultaneously chooses the (set of) harvest effort level(s) that maximizes private profit to that player, taking as given the management scenario and harvest effort levels chosen by the other players.

We modeled a total of four different scenarios for high seas management: Two scenarios when the high seas are open to fishing and two scenarios when the high seas are closed to fishing. Within each scenario, a large range of parameter values were considered.

When the high seas are open to fishing, we considered two scenarios: one that limits harvest in the high seas to the countries whose EEZs are traversed by the stock, and a second scenario that allows open access high seas harvest by an unlimited number of fishermen. In the latter scenario, the high seas represent a true commons, open to all and owned by none, thus the stock in the high seas is expected to be harvested down to the zero marginal profit density set by θ (though in the EEZs it will not be as heavily exploited). Taking this as given, each of the N players chooses a harvest effort level for its EEZ that maximizes the player's profit, given the decisions by the other players. We termed the solution "HS open (OA)". In the other high seas open scenario, each player has two choice variables: harvest effort in its EEZ and harvest effort in the high seas. The choices are determined concurrently and simultaneous among all players. We termed the solution "HS open (N)" to indicate that N players are competing on the high seas.

When the high seas are closed to fishing, we considered two scenarios: one in which the players comply with the high seas closure, and a second scenario in which compliance with the closure is imperfect (i.e., there is poaching in the high seas). In the second scenario we considered positive harvest levels in the high seas by the players in the system, up to a maximum level equal to that found in the scenario HS open (N). The level of compliance was mediated by $0 \leq C \leq 1$, in which harvest in the high seas by a player is $(1 - C)$ multiplied by the player's harvest level in the high seas under HS open (N). Thus setting C=1 allows for no poaching (100% compliance), and setting $C = 0$ returns the solution to HS open (N). Taking C as given, the players choose harvest effort levels in their respective EEZs to maximize their private profits. Note, when $C > 0$ profit

from poaching the high seas is included in each player's choice of EEZ harvest level. For the baseline analyses presented in the main text we set C=1; in the SI we show implications of closing the high seas across the full range of compliance values $0 \leq C \leq 1$.

References

1. Schaefer MB (1957) A Study of the Dynamics of the Fishery for Yellowfin Tuna in the Eastern Tropical Pacific Ocean. Bulletin of the Inter-American Tropical Tuna Commission 2: 247-285.
2. Myers RA, Bowen KG, Barrowman NJ (1999) Maximum reproductive rate of fish at low population sizes. Canadian Journal of Fisheries and Aquatic Sciences 56: 2404-2419.
3. Block BA, Jonsen ID, Jorgensen SJ, Winship AJ, Shaffer SA, et al. (2011) Tracking apex marine predator movements in a dynamic ocean. Nature 475: 86-90.
4. Block BA, Teo SLH, Walli A, Boustany A, Stokesbury MJW, et al. (2005) Electronic tagging and population structure of Atlantic bluefin tuna. Nature 434: 1121-1127.
5. Lehodey P, Senina I, Murtugudde R (2008) A spatial ecosystem and populations dynamics model (SEAPODYM) - Modeling of tuna and tuna-like populations. Progress in Oceanography 78: 304-318.
6. Clark CW (1990) Mathematical Economics: the Optimal Management of Renewable Resources. New York: John Wiley & Sons. 386 p.
7. White C, Kendall BE, Gaines S, Siegel DA, Costello C (2008) Marine reserve effects on fishery profit. Ecology Letters 11: 370-379.
8. Sumaila UR, Khan A, Watson R, Munro G, Zeller D, et al. (2007) The World Trade Organization and global fisheries sustainability. Fisheries Research 88: 1-4.
